# The Effect of Traumatic Brain Injury on Alzheimer’s Disease and Cognitive Decline in Veterans and Non-Veterans

**DOI:** 10.1093/geroni/igab046.1184

**Published:** 2021-12-17

**Authors:** Igor Akushevich, Arseniy Yashkin, Stanislav Kolpakov Nikitin, Julia Kravchenko

**Affiliations:** 1 Duke University, Durham, North Carolina, United States; 2 Duke University, Morrisville, North Carolina, United States

## Abstract

We assess the differences in the effect of traumatic brain injury (TBI) on the decline in cognitive status and the risk of Alzheimer’s disease and related dementia (AD/ADRD) between veteran and non-veteran respondents of the Health and Retirement Study (HRS) and measure the sensitivity of these differences to the incremental introduction of controls for associated risk factors. Three groups of AD/ADRD risk-related variables were used: i) demographic/socioeconomic factors, including gender, race, marital status, education, income, and the number of limitations in activities of daily living; ii) comorbidities, including co-existing depression/post-traumatic stress syndrome (PTSD), substance (alcohol, tobacco and/or prescription drug) abuse, diabetes mellitus, stroke, and heart failure; and iii) genetic factors, including the presence of at least one pair of the APOE4 allele and a series of polygenic risk scores associated with AD hallmarks. The dynamics of changes in cognitive impairment in response to TBI, PTSD, and mild cognitive impairment were validated against respective measures estimated using the Department of Defense Alzheimer’s Disease Neuroimaging Initiative (DoD-ADNI) data. The results of the analyses showed that TBI and PTSD were strongly associated with cognitive decline and the risks of AD/ADRD in both veteran and non-veteran subpopulations in HRS data and the difference between them was not statistically significant. Effect magnitude decreased with the addition of risk-related control variables but remained associated with the increased risks. Prevalence of mild cognitive impairment was associated with TBI at baseline in DoD-ADNI data, but no cognitive decline was observed during one year of follow-up.

